# Gut Bacterial Diversity of Insecticide-Susceptible and Insecticide-Resistant *Megalurothrips usitatus* (Thysanoptera: Thripidae) and Elucidation of Their Putative Functional Roles

**DOI:** 10.3390/insects14080669

**Published:** 2023-07-27

**Authors:** Bifeng Zhu, Yueyin Chen, Chenyan Zhou, Haolong Li, Shaukat Ali, Jianhui Wu

**Affiliations:** 1 Engineering Research Center of Biological Control, Ministry of Education and Guangdong Province, South China Agricultural University, Guangzhou 510642, China; 20212022024@stu.scau.edu.cn (B.Z.); scauzcy@stu.scau.edu.cn (C.Z.); 20222022012@stu.scau.edu.cn (H.L.); 2 Meizhou Depot of Guangdong Grain Reserve Management Group Co., Ltd., Meizhou 514071, China; cyy13414969523@163.com

**Keywords:** *Megalurothrips usitatus*, insecticide resistance, gut bacterial community, Illumina MiSeq, functional roles

## Abstract

**Simple Summary:**

In this study, the researchers investigated the role of gut bacteria in an insect pest called *Megalurothrips usitatus* and its resistance to an insecticide and an entomopathogenic fungus. They compared the gut bacterial communities of an insecticide-susceptible population and an insecticide-resistant population of *Megalurothrips usitatus*. Through High-throughput sequencing, they found that the insecticide-resistant population exhibited significantly higher resistance to the insecticide and the *Beauveria brongniartii*. The sequencing also revealed changes in the abundance of specific bacterial species in response to the insecticide and the *Beauveria brongniartii*. The researchers observed that the gut bacteria of *Megalurothrips usitatus* are primarily involved in various metabolic activities, including the degradation of the *Beauveria brongniartii*. Some of these bacteria may even reduce the virulence of the fungus, thereby weakening its biocontrol ability. The results of this study provide a basis for future elucidation of the role of intestinal bacteria in the symbiotic relationship of insecticide resistance and the design of new management strategies for *Megalurothrips usitatus* using *Beauveria brongniartii* for biological control.

**Abstract:**

The gut bacterial microbiota of insects plays a crucial role in physiological, metabolic, and innate immune processes. In the current study, the gut bacterial communities of an insecticide-susceptible (IS), and a resistant (IR) population of a major legume pest, *Megalurothrips usitatus (Bagnall)*, were evaluated. The 16S rDNA V3 + V4 regions of *M. usitatus* infected with *Beauveria brongniartii* along with the intestinal flora of both populations were sequenced based on a High-throughput sequencing platform. Toxicological bioassays revealed that the IR population exhibited resistance to acetamiprid and *B. brongniartii* isolate SB010 at levels of 138.0-fold and 55.6-fold higher, respectively, compared to the IS population. Through 16S High-throughput sequencing, the results indicate that both resistant populations, as well as *B. brongniartii* infestation, reduce the number of species of *M. usitatus* gut microbes. Using KEGG function prediction, it was found that most intestinal bacteria were involved in various metabolic activities, and the abundance of resistant populations was higher than that of sensitive populations. The bacteria in the gut of *M. usitatus* are mainly involved in various metabolic activities to achieve the degradation of *B. brongniartii*. This study provides valuable insights into the interaction between gut bacteria, insecticide resistance, and *Beauveria. brongniartii* infection in *Megalurothrips usitatus*, which can help inform future pest control strategies.

## 1. Introduction

*Megalurothrips usitatus* (Bagnall) (Thysanoptera: Thripidae), is one of the most economically important pests of *Vigna unguiculata* (L.) Walp in South China. It is an omnivorous pest which is mainly a threat to legume crops [1]. It damages cowpeas throughout the reproductive period, with its filing sucking mouthparts that feed on the plant’s leaves, flowering organs, pods, and other young tissue sap. In severe cases, the entire plant withers, resulting in substantial economic losses [2]. More importantly, *M. usitatus* is capable of transmitting plant viruses such as *Tobacco streak virus* (TSV) and also *Peanut bud necrosis virus* (PBNV) [3,4,5]. Therefore, the necessity to have efficient alternative control strategies is crucial for the effective management of *M. usitatus*. Currently, chemical control is the main method to control *M. usitatus* [6,7]. Due to the characteristics of *M. usitatus*, such as high concealment, fast reproduction, and short generation cycles, the use of chemical agents for effective field control has become common. However, the irregular use of pesticides can cause high resistance to many chemical agents and also environmental pollution [8]. *Beauveria brongniartii* is an efficient biological agent for farm and forestry invertebrate pest control. It is widely used across farmland in China because it is listed as environmentally friendly, harmless to animals and humans, and offers continuous effective control [9]. *Beauveria brongniartii* (Sacc.) Petch is a promising fungal pathogen, which is virulent against different insect species [10].

Endosymbionts inhabiting the gut of insects can provide many benefits to their hosts. These include aiding in food digestion, reproduction, immunity, tissue homeostasis, adaptation to the environment, and resistance to pathogens and pesticides [11,12,13]. Recent studies have shown a direct relationship between insecticide resistance development and the gut microbiome of insects [14,15]. In a recent study, *Citrobacter freundii*, an intestinal bacterium of the oriental fruit fly, was found to degrade the organophosphorus trichlorfon to chloral hydrate and dimethylphosphate, resulting in trichlorfon resistance [16]. These endosymbionts are also known to affect the virulence of insect pathogenic fungi [17]. Pea aphids harboring *Regiella insecticola* Moran (Enterobacterales: Enterobacteriaceae) have been reported to exhibit increased resistance against infection by the entomopathogenic fungus *Pandora neoaphidis* (Entomophthorales: Entomophthoraceae) [18]. Similarly, *R. insecticola*, *Rickettsia* sp., and *Rickettsiella* sp. reduced the mortality of their host aphids and also decreased the sporulation of fungus on dead aphids [19]. *R. insecticola* provided resistance against the aphid-specific fungal pathogen *Zoophthora occidentalis*, while no resistance against a generalist fungal entomopathogen such as *Beauveria bassiana* was reported [20]. Based on the above mentioned facts, dynamic changes in endosymbionts of resistant and susceptible insect populations treated with insect pathogenic fungi will be worth studying to further elucidate the role of endosymbionts in insects’ immune systems.

In this study, we used ultra-deep sequencing of the V3 hypervariable region of the 16S rRNA gene to investigate the composition and diversity of the gut bacteria associated with *M. usitatus* infested with *B. brongniartii* isolate SB010. We provide a comparative catalog of gut bacteria associated with insecticide-susceptible (IS) and insecticide-resistant (IR) populations of *M. usitatus*. We discuss variations in bacterial community structure and their potential roles in imparting insecticide resistance to the host. Additionally, we performed putative functional profiling based on taxonomic diversity, gaining preliminary insights into the symbiotic relationship between *M. usitatus* and its gut bacteria. 

## 2. Materials and Methods

### 2.1. Insect Populations

*M. usitatus* collected from cowpea growing areas in Haikou, China, in 2017 was brought back to the Engineering Research Center of the Ministry of Education for Biological Control, South China Agricultural University, and placed in a climate control chamber at 26 ± 1 °C, 70 ± 5% relative humidity (RH), and a 16:8 h (light/dark) photoperiod to rear and breed *M. usitatus* with bean pod, during which time it was not exposed to any pesticides in preparation for subsequent screening of susceptible and resistant populations.

*M. usitatus* from continuous climate control chamber breeding for four years and not exposed to any pesticides were used as insecticide-susceptible (IS) populations. After two years of the climate control chamber breeding and rearing, a resistance screening was conducted based on the first generation of acetamiprid solution configured according to the results of the bioassay, and the concentration was increased by 200 mg/L for each screening and the population that had been screened for resistance for two years and 32 generations was considered as the insecticide-resistant (IR) population.

### 2.2. Insecticides and Toxicological Bioassay

To determine the resistance status of the two populations of *M. usitatus*, *B. brongniartii* isolate SB010 and acetamiprid were used. *B. brongniartii* isolate SB010 was cultured on Potato Dextrose Agar (PDA) with the plates incubated at 26 °C in complete darkness for 10 days until the harvesting of conidia. This was undertaken by adding 10 mL of sterile 0.05% Tween 80 to the culture and scraping the conidia with a sterile spatula.

To assay the dose response of *M. usitatus* to the insecticides, different concentrations of *B. brongniartii* (1 × 10^8^,1 × 10^7^, 1 × 10^6^, 1 × 10^5^, 1 × 10^4^ conidia.mL^−1^) and acetamiprid (4800, 2400, 1200, 600, 300 mg.L^−1^) insecticides were used for the experiment. Bean pods and Centrifuge tubes treated with sterile ddH2O served as a control.; 20 *M. usitatus* adults were treated at each concentration and the process was repeated three times. A 15 mm × 75 mm flat-bottomed test tube was soaked in the prepared spore suspension for 2 h, allowed to air-dryand then prepared for use. At the same time, fresh cowpeas were cut into 1 cm pieces without holes at both ends, soaked in the spore suspension for 15 s and then removed. The cowpeas were then dried and placed in the flat-bottomed test tubes as mentioned above. The adult *M. usitatus* were placed in the RXZ-500C intelligent artificial climate, and their mortality was recorded over the following 3 days. 

Mortality data were subjected to probit analysis using SPSS21.0 to determine the lethal concentration (LC_50_) values and their 95% confidence limits (CLs). Levels of resistance were classified based on the resistance factor (RF) at LC_50_: RF < 10-fold indicated low resistance, RF = 10–40-fold indicated moderate resistance, RF = 40–160-fold indicated high resistance, and RF > 160-fold indicated extremely high resistance. RF was calculated as the ratio of LC_50_ between insecticide-resistant (IR) and insecticide-susceptible (IS) populations.

### 2.3. Extraction of DNA from the Guts of the IS and IR M. usitatus

A 15 mm × 75 mm flat-bottomed test tube was soaked in 1 × 10^7^ conidia.mL^−1^ spore suspension for 2 h, dried naturally, and prepared for use. Meanwhile, fresh cowpeas were cut into 1 cm sections without holes at both ends, soaked in spore suspension for 15 s, removed, dried, and placed in the above flat-bottomed test tube, and *M. usitatus* adults were released into the flat-bottomed test; the tubes were then sealed with cotton, and placed at 26 ± 1 °C and 12 L:12 D in a lighted incubator. Sterile water treatment with 0.05% Tween-80 was used as a blank control. Three replicates of each treatment were set up, and the surviving test worms were collected following 1 day, 2 days, and 3 days. Their abdomens were cut under aseptic conditions and sent to Guangzhou Kidio Biotechnology for bacterial 16S High-throughput sequencing. The sensitive control was recorded as S-CK, and the resistance control was recorded as R-CK. The 1 × 10^7^ conidia.mL^−1^-treated sensitive populations were recorded as B1, B2, and B3 at 1 day, 2 days, and 3 days, respectively, 1 × 10^7^ conidia.mL^−1^-treated resistance populations were recorded as D1, D2, and D3 at 1 day, 2 days, and 3 days, respectively. The DNA was individually extracted from the adult guts of each population using the HiPure Stool DNA Kits (Magen, Guangzhou, China) following the manufacturer’s instructions. The extracted DNA was assessed for quality using electrophoresis on a 1% agarose gel and stained with ethidium bromide, following the manufacturer’s protocol. The amplification products from the second round were purified using AMPure XP Beads. The extracted DNA was stored at −80 °C. DNA from adults of each population was pooled, resulting in eight samples selected for amplicon library construction.

### 2.4. Amplicon Library Construction and Illumina Sequencing

Extraction of DNA from the intestinal bacteria of *M. usitatus* was performed using the HiPure Stool DNA Extraction Kit according to the instructions to take an appropriate amount of sample DNA in a centrifuge tube and dilute the sample to 1 ng/μL using sterile water. In this experiment, the 16S rDNA V3-V4 region was selected as the target region for amplification. Using diluted genomic DNA as the template, universal primers 341F and 806R with barcode (341F: CCTACGGGNGGCWGCAG, 806R: GGACTACHVGGGTATCTAAT [21], amplification of collected DNA. Amplification conditions: pre-deformed at 94 °C for 2 min, denatured at 98 °C for 10 s, annealing at 62 °C for 30 s, extended at 68 °C for 30 s for 30 cycles, and finally at 68 °C for 5 min. The PCR reaction was performed in triplicate. Amplification system: 50 μL mixture consisting of 5 μL 10 × KOD buffer, 5 μL 2 mM dNTPs, 3 μL 25 mM MgSO4, 1.5 μL upstream and downstream primers (10 μM), 1 μL KOD polymerase, 100 ng template DNA. PCR-related reagent manufacturers (TOYOBO, Tokyo, Japan). The amplicon was collected from 2% agarose gel and purified using an AxyPrep DNA gel extraction kit (Axygen Biosciences, Union City, CA, USA) according to the manufacturer’s instructions. ABI StepOnePlus real-time PCR system (Life Technologies, Foster City, CA, USA) was used to determine the dosage. The purified amplicon was sent to Guangzhou Gidio Biotechnology Co., Ltd. (Guangzhou, China) for double-ended sequencing (PE250) on the Illumina platform according to standard procedures.

### 2.5. Processing of the Sequence Reads and Data Analysis

Raw data from the Illumina platform were filtered using FASTP [22] and the clean reads obtained after filtering were used for assembly analysis. The clean reads were merged into tags using FLSAH [23] with a minimum overlap of 10 bp and a mismatch rate of up to 2% threshold. Based on the filtering conditions outlined in the literature [24], the QIIME process was used. The tags were intercepted according to the quality control process of QIIME [25], and the intercepted tags data set was further filtered for tags with consecutive high-quality bases less than 75% of the length of the tag. Based on the reference database, chimera checking of tags was performed using the UCHIME algorithm [26]. The clean tag obtained after filtering the chimeras was used for subsequent analysis. 

The clean tags were clustered into Operational Taxonomic Units (OTUs) by ≥ 97% similarity using the UPARSE [27] process. The tag sequences with the highest abundance were selected as representative sequences for each OTU. The intergroups sharing unique OTUs were analyzed separately for Venn using the R language VennDiagram package [28]. The OTU representative sequences were compared to the SILVA [29] database or Greengene [30] database or UNITE [23] database or ITS2 [31] database for species classification annotation using the RDP annotation software [32] with a confidence threshold set at 0.8 to 1. A confidence threshold of 0.8 to 1 was set using Krona [33] to display abundance statistics for each species classification. Species abundance stacked maps were displayed using the R language ggplot2 package [34]. Species abundance heat maps were plotted using the R language pheatmap package [35]. Pearson correlation analysis of species was calculated using the psych package. Species correlation network plots were generated using the Omicsmart dynamic real-time interactive online data analysis platform (http://www.omicsmart.com (accessed on 16 May 2021)) or the R language igraph package. KEGG (Kyoto Encyclopedia of Genes and Genomes) metabolic pathway analysis of samples was performed using Tax4Fun [36].

## 3. Results

### 3.1. Toxicological Bioassay

Table 1 presents the LC_50_ values of the insecticide-susceptible (IS) and insecticide-resistant (IR) populations for the tested insecticides. The LC_50_ values of the IR population for *B. brongniartii* isolate SB010 and acetamiprid showed significant variations compared to the IS population. The RF values indicated that the IR population exhibited high levels of resistance to acetamiprid (RF = 138.04) and *B. brongniartii* (RF = 55.62). However, the resistance level to acetamiprid in the two populations of *M. usitatus* was 2.5 times higher than that of *B. brongniartii* SB010.

### 3.2. Illumina MiSeq Metagenomic Data and Taxonomic Assignments

The Illumina Miseq high-throughput sequencing platform was used to sequence the 16S rDNA V3–V4 regions of the IS and IR populations. The IS and IR populations were treated with 1 × 10^7^ conidia/mL for 1 day, 2 days, and 3 days. The original *M. usitatus* bands were 102,074~109,466, which were optimized to obtain the final splice yield of 92,964~99,172. The quality control efficiency was maintained at 73~76%. The OTU clustering analysis was performed using USEARCH software on the valid bands of all samples, and the sequences were clustered into OTUs with 97% identity. A total of 846 OTUs were obtained for the eight samples. The number of OTUs obtained for each treatment is shown in Table 2. Based on the OUT taxonomic status, a total of 10 Phyla, 10 Orders, 10 Families, 10 Genera, and 10 Species were identified.

**Figure 1:**
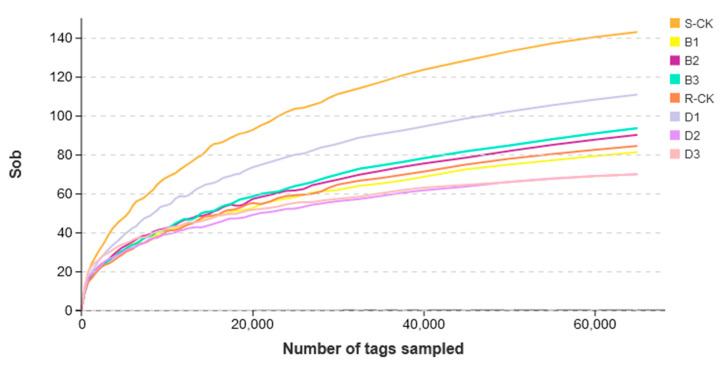
*Megalurothrips usitatus* sample dilution graph.

The dilution curve of the samples showed that the number of species increased abruptly with the increase of the sampling volume in the early stage, before plateauing at the end. The increase of the sampling volume no longer changed the number of species significantly (Figure 1). The results demonstrate that the majority of the microorganisms were included in the sequencing; the sequencing volume was sufficient, the sequencing depth met the experimental requirements, and the experimental analysis was scientifically reliable.

**Figure 2:**
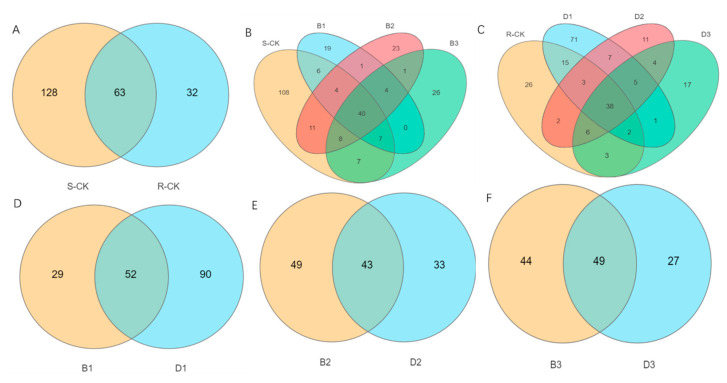
Comparison of Venn diagrams between two populations. Notes: (**A**) represents the Venn diagram of sensitive versus resistant populations control; (**B**) represents the Venn diagram of sensitive populations control versus sensitive populations treated; (**C**) represents the Venn diagram of resistant populations control versus resistant populations treated; (**D**) represents Venn diagram of *B. brongniartii* treated sensitively versus resistant populations 1st; (**E**) represents Venn diagram of *B. brongniartii* treated sensitively versus resistant populations 2nd; (**F**) represents Venn diagram of *B. brongniartii* treated sensitively versus resistant populations 3rd.

The OUTs of all samples obtained were classified and the number of OTUs unique and common to each sample was analyzed by plotting Venn diagrams. As shown in Figure 2, S-CK had 191 species and 128 endemics, while R-CK had 95 species and 32 endemics, with 63 identical species between them (Figure 2A). The number of B1 species decreased significantly after inoculation of sensitive populations with *B. brongniartii* isolate SB010, with only 81 species, of which 24 were endemic; the proportion of B2 and B3 endemic species did not change significantly (Figure 2A). The proportion of species-specific to B2 and B3 did not change significantly (Figure 2B). After inoculation of the resistant populations with *B. brongniartii* isolate SB010, unlike B1, The number of D1 species increased sharply to 142 instead of decreasing, of which, 84 were endemic; the number of D2 species decreased sharply to 76, with a consequent reduction in the number of endemic species to 27; the proportion of species in D3 remained unchanged (Figure 2C).

An independent comparison of the treatments of the two populations showed that D1 had significantly more endemic species than B1; 52 species were present between them. D2 had fewer endemic species than B2, with 43 species in common between them. D3 and B3 did not differ from B2 and D2 in terms of the number of endemic and shared species (Figure 2D–F). It can be seen that resistance to acetamiprid reduced the diversity of the *M. usitatus* gut flora and that *B. brongniartii* isolate SB010 treatment also reduced the diversity of the *M. usitatus* gut flora. The difference was that the pathogen infestation directly reduced the diversity of the intestinal microflora of the sensitive populations, whereas, in the case of the acetamiprid-resistant populations, the diversity of the intestinal flora was first sharply up-regulated at 1 day and then reduced after 2 days. 

**Figure 3:**
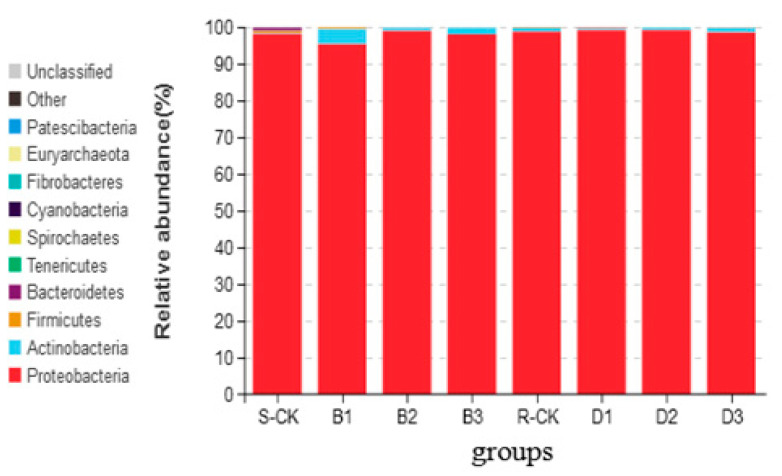
Abundance analysis of *Megalurothrips usitatus* at Phylum level for different treatments.

OTU abundance analysis was performed for each treatment of the two populations of *M. usitatus*. Based on the fact that the majority of OTUs could not be identified at the species level, then abundance analysis was chosen at the Phylum and Genus level in this study. The results showed that at the Phylum level, Proteobacteria, Bacteroidetes, and Firmicutes dominated the sensitive populations without *B. brongniartii* infection. However, uninfected resistant populations are mainly Proteobacteria and Actinobacteria. After *B. brongniartii* infection, Proteobacteria and Actinomyces dominated the two populations within 1–3 days, with Proteobacteria taking the dominant position (Figure 3).

**Figure 4:**
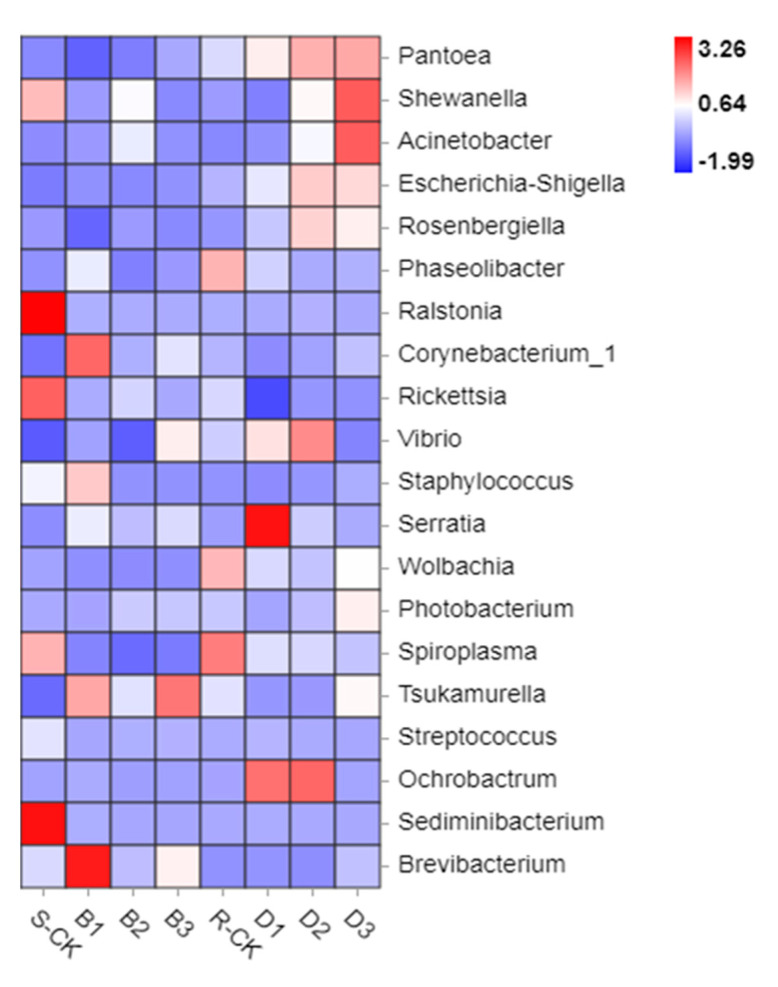
Abundance heat maps of species under different treatments.

At the Genus level, before treatment, compared to sensitive populations, the abundance of resistant populations of *Pantoea*, *Escherichia-Shigella*, *Phaseolibacter*, *Vibrio*, *Wolbachia,* and *Tsukamurella* increased. The abundance of *Shewanella*, *Ralstonia*, *Rickettsia*, *Staphylococcus,* and *Sediminibacterium* decreased. Compared with susceptible populations, local infection of *B. brongniartii* resulted in progressive colonization of *Panthenium*, *Shivarella*, *Acinetobacter*, *Eschella*, *Rosenbergiella*, *Wolbachia*, and *Fasciculla* in resistant populations. *Panthenia*, *Eschella*, and *Wolbachia* were over-propagated in resistant populations of infected *B. brongniartii* but were not dominant in susceptible populations of infected *B. brongniartii* (Figure 4).

### 3.3. Functional Analysis of Intestinal Bacteria of Two Populations of M. usitatus after Infection with B. brongniartii Isolate SB010

**Figure 5:**
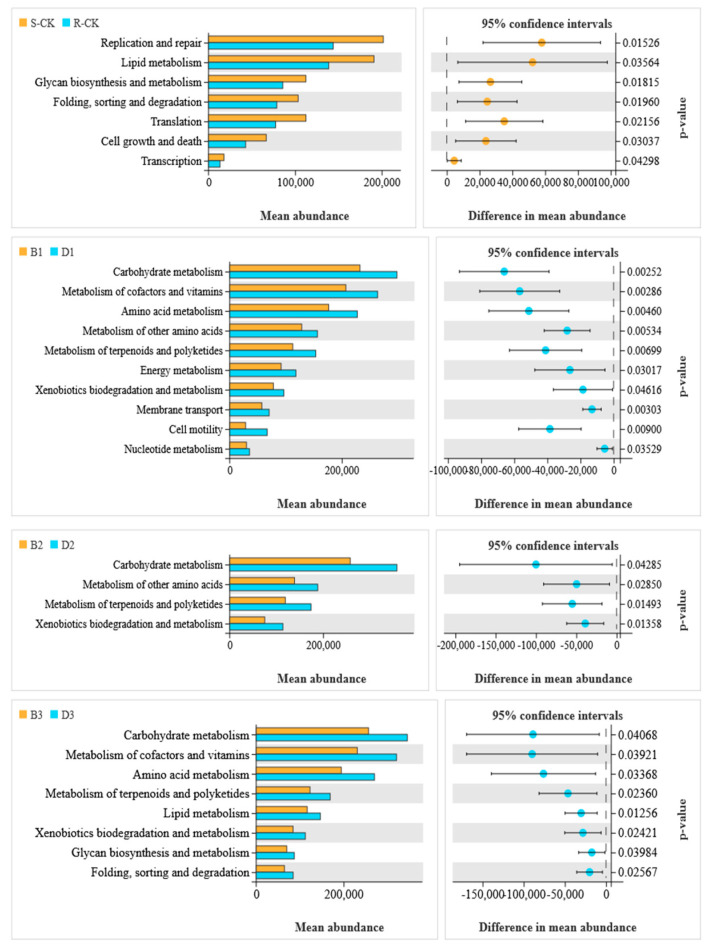
KEGG pathway analysis of *Megalurothrips usitatus* by treatment.

PICRUSt2 software(2.0) was used in combination with the KEGG database to functionally enrich the gut microbes of *M. usitatus* and to screen for representative functional pathways that differed between groups. The results showed that, before treatment with *B. brongniartii*, the pathways involved in replication and repair, lipid metabolism, glycan biosynthesis, metabolism, folding, sorting and degradation, and cell growth and death, all displayed a higher number of sensitive populations than R-resistant populations. Following *B. brongniartii* treatment, the abundance of intestinal bacteria involved in the differential pathways was higher in the resistant populations than in the sensitive populations. Equally, carbohydrate metabolism, metabolism of cofactors and vitamins, amino acid metabolism, metabolism of terpenoids and polyketides, and xenobiotic biodegradation and metabolism were all more abundant on day 1 (Figure 5). The abundance of bacteria in carbohydrate metabolism, metabolism of other amino acids, xenobiotic biodegradation, and in metabolism was higher after 2 days. The abundance of bacteria in the metabolism of other amino acids, xenobiotic biodegradation and metabolism, glycan biosynthesis, and metabolism was higher on the 3rd day (Figure 5). Among them pathways, the xenobiotic biodegradation and metabolism, carbohydrate metabolism, and amino acid metabolism were present in good abundance in both lines within 3 days of *B. brongniartii* treatment in both populations; but were higher in the resistant populations.

## 4. Discussion

Insect gut microbes play an important role in insect feeding and resistance to unfavorable foreign conditions. To date, various aspects of insect gut microorganisms have been studied. Some studies suggested that sublethal doses of imidacloprid did not affect the bacterial community structure in the midgut of *Apis mellifera ligustica* [37]. Wenhao Li [38] found that resistance to Nitenpyram significantly increased the richness and uniformity of the intestinal microbial community of *Nilaparvata lugens*. However, other studies have shown that treatments with *Beauveria bassiana*, Avermectin, Tetrachlorantraniliprole, Betacypermethrin, and Chlorantraniliprole can reduce intestinal microbial diversity and evenness of *Grapholitha molesta* and *Cydia pomonella* [39]. Zhao Tianyu [40] also found that the richness and evenness of intestinal flora of the larvae of Bifenthrin-resistant strains of *Ectropis obliqua* were lower than those of sensitive strains. The present study is the first to investigate the cause of resistance in *M. usitatus* from the perspective of gut bacteria. In this study, we found that the gut diversity of the resistant populations was lower than that of the sensitive populations and that the infestation of both *M. usitatus* populations with *B. brongniartii* isolate SB010 also led to a decrease in gut microbial diversity. The reasons for such different results from different experiments could be either the different subjects investigated or the different agents treated, both of which are key factors that can cause changes in the intestinal flora of insects.

*Rickettsia* is an insect secondary commensal bacteria that can regulate reproductive behavior, favorably affect insect growth and development, and is also known to enhance insect susceptibility to insecticides [41,42,43,44]. Studies have shown that infection with *Rickettsia* can make *Bemisia tabaci* more sensitive to insecticides such as Acetamidine, Thiamethoxam, and Imidacloprid, and the higher the sensitivity of *Bemisia tabaci* to insecticides, the higher the relative density of *Rickettsia* [45]. A large number of studies have shown that the proliferation of *Pantoea* can degrade various toxins, including pesticides (Acephate, Pyrethroids, Isothiocyanate) and pathogenic bacteria (*Beauveria bassiana*, *Metarhizium anisopliae*), thereby reducing their virulence [46,47,48,49]. Studies have shown that *Wolbachia* can increase the resistance of *Bemisia tabaci* to Acetamiprid, Drosophila to RNA viruses such as *dengue virus* and *Drosophila C virus*, and can also increase the resistance of *Drosophila* melanogaster to *Beauveria bassiana* [50,51,52,53]. *Rosenbergiella* was originally present in pollen nectar, and three new strains of Rosenbergiella were subsequently isolated from nectar, and the bacteria were acquired by insects from nectar feeding [54,55]. The bacterium belongs to Enterobacteriaceae, which has the function of resisting pathogenic bacteria, degrading foreign harmful substances, enhancing host adaptability, and promoting host carbon and nitrogen metabolism cycles [56]. Acinetobacter can participate in biological nitrogen removal and intestinal nutrient metabolism. Studies have found that Acinetobacter in the intestine of Cabbage root fly larvae (*Delia radicum*) can degrade Isothiocyanates. *Acinetobacter calcoaceticus* can effectively degrade Pyrethroids insecticides [57,58].

In this experiment, Pantobacterium, Escherichia, Phasobacter, Vibrio, Wolbachia, Bunchura, Shiwanella, Solanacearia, Rickettsiella, Staphylococcus and Sediminibacterium have changed in abundance in the two populations. The abundances of Rickettsia, Panthenia, and Wolbachia are consistent with the results of previous studies, and the results are reliable. Therefore, in addition to the three symbiotic bacteria mentioned above, the remaining endosymbiotic bacteria may also be associated with *M. usitatus* resistance. After *B. brongniartii* isolate SB010 infection, Pantobacter, Shiwanella, Acinetobacter, Escherichia, Roxella, Wolbachia, and Bunchura gradually colonized in resistant populations compared with sensitive populations. In addition to Wolbachia and Pantobacter, which have been studied, other endosymbiotic bacteria may also be related to reducing the virulence of *B. brongniartii*.

The present study also annotated both intestinal microbes functionally by KEGG and found that after *B. brongniartii* isolate SB010 infestation, the majority of bacteria were clustered into various metabolic pathways, including foreign body metabolism, lipid metabolism, and energy metabolism. This suggests that these bacteria are responding to *B. brongniartii* isolate SB010 infestation through these metabolic pathways. It has been found that microorganisms can counteract adverse external influences and provide host insects with favorable conditions to cope with proportional external environments through energy supplementation [59,60]. It can be seen that these bacteria in the gut of *M. usitatus* mainly achieve degradation of *B. brongniartii* through various metabolic activities, so as not to be harmed by it.

## 5. Conclusions

In summary, by utilizing deep sequencing to analyze the V3 hypervariable region of the 16S rRNA gene, we have created a comparative inventory of bacterial communities within the gut of *B. brongniartii*-infected *M. usitatus* populations, distinguishing between IR and IS populations. The bacteria associated with the gut of *M. usitatus* exhibit a wide range of capabilities, thereby contributing to various fitness characteristics of the host. It was observed that the IR populations harbor a diverse array of bacterial communities, with a notable enrichment of bacteria capable of performing functions related to detoxification. To comprehensively comprehend the potential involvement of symbionts in insecticide detoxification, it is crucial to conduct thorough functional analyses. Such investigations will pave the way for innovative strategies in insect pest control by manipulating gut bacteria. This study serves as an initial endeavor in establishing an all-inclusive inventory of gut bacteria present in *M. usitatus*, employing deep-sequencing methods. The bacteria identified in this study serve as a foundational platform for future research endeavors aimed at exploring symbiont-based strategies for the effective management of insecticide resistance in *M. usitatus*.

## Figures and Tables

**Table 1:** Dose-responses of two populations of *Megalurothrips usitatus* to various insecticides.

Insecticide	Population	Slope ± SE	LC_50_ (95% CL) ^a^	LCR_50_ (95%CL) ^b^
Acetamiprid-	IS	1.00 ± 0.18	8.63 (4.18–10.57)	138.04 (172.24–186.48) *
	IR	1.57 ± 0.17	1191.28 (719.97–1971.14)	
*B. brongniartii*	IS	0.38 ± 2.70	1.2 × 10^6^ (2.5 × 10^5^–6.0 × 10^6^)	55.62 (10.26–30,150.03) *
	IR	0.31 ± 2.56	6.8 × 10^7^ (2.6 × 10^6^–1.8 × 10^9^)	

^a^ The LC_50_ values are expressed as mg of a.i./L for acetamiprid, and ,The LC_50_ values are expressed as conidia of a.i.mL for *B. brongniartii*. ^b^ The lethal concentration ratios at LC_50_, along with their 95% CL, were calculated using SPSS21.0 software. * The LCR_50_ is considered statistically significant at a significance level of α = 0.05 if the 95% CL does not include the value of 1.0.

**Table 2:** 16S rDNA sequencing results and OTU number statistics for each treatment of *Megalurothrips usitatus*.

Sample	Raw Tags	Clean Tags	Effective Tags	Effective Ratio (%)	OTUs (Individual)
S-CK	102,074	96,541	92,964	74	191
B1	109,466	105,369	99,172	74	81
B2	109,422	104,815	98,539	73	92
B3	104,250	100,345	94,579	74	93
R-CK	106,162	101,810	95,872	73	95
D1	105,975	102,716	98,807	76	142
D2	102,590	98,920	93,522	74	76
D3	103,351	99,454	94,125	74	76

## Data Availability

The raw data supporting the conclusions can be made available by the corresponding author on request.
